# Image-guided and passively tumour-targeted polymeric nanomedicines for radiochemotherapy

**DOI:** 10.1038/sj.bjc.6604561

**Published:** 2008-09-02

**Authors:** T Lammers, V Subr, P Peschke, R Kühnlein, W E Hennink, K Ulbrich, F Kiessling, M Heilmann, J Debus, P E Huber, G Storm

**Affiliations:** 1 Department of Innovative Cancer Diagnosis and Therapy, Clinical Cooperation Unit Radiotherapeutic Oncology, German Cancer Research Center, Im Neuenheimer Feld 280, 69120 Heidelberg, Germany; 2 Department of Pharmaceutics, Utrecht Institute for Pharmaceutical Sciences, Utrecht University, Sorbonnelaan 16, 3584 CA Utrecht, The Netherlands; 3 Institute of Macromolecular Chemistry, Academy of Sciences of the Czech Republic, Heyrovsky Square 2, 162 06 Prague 6, Czech Republic; 4 Department of Medical Physics in Radiology, Junior Group Molecular Imaging, German Cancer Research Center, Im Neuenheimer Feld 280, 69120 Heidelberg, Germany; 5 Department of Biomedical In vivo Imaging, Institut Curie – Centre de Recherche, and INSERM U.759, Orsay, France; 6 Department of Radiooncology and Radiotherapy, Heidelberg University, Im Neuenheimer Feld 110, 69120 Heidelberg, Germany

**Keywords:** drug targeting, nanomedicine, polymer therapeutics, radiotherapy, chemotherapy, combined modality therapy

## Abstract

Drug targeting systems are nanometer-sized carrier materials designed for improving the biodistribution of systemically applied (chemo-) therapeutics. Reasoning that (I) the temporal and spatial interaction between systemically applied chemotherapy and clinically relevant fractionated radiotherapy is suboptimal, and that (II) drug targeting systems are able to improve the temporal and spatial parameters of this interaction, we have here set out to evaluate the potential of ‘carrier-based radiochemotherapy’. *N*-(2-hydroxypropyl)methacrylamide (HPMA) copolymers were used as a model drug targeting system, doxorubicin and gemcitabine as model drugs, and the syngeneic and radio- and chemoresistant Dunning AT1 rat prostate carcinoma as a model tumour model. Using magnetic resonance imaging and *γ*-scintigraphy, the polymeric drug carriers were first shown to circulate for prolonged periods of time, to localise to tumours both effectively and selectively, and to improve the tumour-directed delivery of low molecular weight agents. Subsequently, they were then shown to interact synergistically with radiotherapy, with radiotherapy increasing the tumour accumulation of the copolymers, and with the copolymers increasing the therapeutic index of radiochemotherapy (both for doxorubicin and for gemcitabine). Based on these findings, and on the fact that its principles are likely broadly applicable, we propose carrier-based radiochemotherapy as a novel concept for treating advanced solid malignancies.

The combination of radiotherapy and chemotherapy has been evaluated extensively in the past few decades (Vokes and Weichselbaum, 1990; Seiwert *et al*, 2007). Besides significantly increasing the efficacy of radiotherapy, however, the simultaneous application of chemotherapy also substantially increases its toxicity (Maduro *et al*, 2003; Rowell and O’rourke, 2004). As external beam radiotherapy can nowadays be delivered with extremely high levels of spatial specificity (Fuks *et al*, 1991; Bernier *et al*, 2004), this is likely mostly due to the low degree of spatial specificity that chemotherapeutic agents generally present upon intravenous (i.v.) administration. Based on this notion, and on the fact that drug targeting systems are known to be able to improve both the temporal (circulation time, tumour residence time) and the spatial (tumour accumulation, tumour-to-organ ratio) parameters of drug therapy (Moses *et al*, 2003; Torchilin, 2005; Duncan, 2006; Lammers *et al*, 2008), we reasoned that the implementation of a drug targeting system might be able to increase the therapeutic index of radiochemotherapy. The rationale for this novel combination regimen, which we have termed ‘carrier-based radiochemotherapy’, relies on the notion that on the one hand, radiotherapy improves drug targeting (i.e. the tumour accumulation of drug targeting systems), and that on the other hand, drug targeting improves radiochemotherapy (i.e., the temporal and spatial interaction between daily radiotherapy and weekly chemotherapy; see Figure 1).

Concerning the former aspect of carrier-based radiochemotherapy, several different mechanisms can be envisioned by which radiotherapy increases the tumour accumulation of drug targeting systems (Figure 1B). Besides reflecting, for instance, on the integrity and function of the tumour vasculature (V), and on the expression of certain cellular receptors (R), it is also known to affect several cell membrane-related (C), nuclear (N), mitochondrial (M) and signalling (S) processes. By eliciting such effects, radiotherapy has been shown to induce (I) an increase in the production of vascular endothelial growth factor (VEGF) (Park *et al*, 2001) and fibroblast growth factor (FGF) (Lee *et al*, 1995), (II) an increase in apoptosis and endothelial cell apoptosis (Garcia-Barros *et al*, 2003), (III) a decrease in tumour cell density (Peschke *et al*, 1999), and (IV) a reduction in interstitial fluid pressure (Znati *et al*, 1996). By means of the former phenomenon, radiotherapy is considered to be able to increase the permeability of the vasculature towards long-circulating nanomedicines (Samaniego *et al*, 1998; Feng *et al*, 1999), and by means of the latter three, it likely improves their penetration and their intratumoral distribution (Jain, 1994; Netti *et al*, 1995; Au *et al*, 2001).

The rationale for the latter aspect of carrier-based radiochemotherapy, that is, for the assumption that drug targeting systems are able to improve the interaction between radiotherapy and chemotherapy, is depicted schematically in Figure 1C. Drug targeting systems are generally designed to be stable in circulation, and to release the conjugated or entrapped active agent only at the target site (Torchilin, 2005; Duncan, 2006; Lammers *et al*, 2008). As a result, as compared to an i.v. applied free drug, the peak plasma concentration of a tumour-targeted agent tends to be reduced, and the degree of systemic toxicity can often be attenuated (Figure 1C; upper two panels). For doxorubicin, for instance, both polymeric (Yeung *et al*, 1991; Duncan, 2006) and liposomal (Ewer *et al*, 2004; Torchilin, 2005) drug targeting systems have been shown to be able to reduce the incidence of cardiomyopathy. At the same time, by increasing the concentration of the active agent at the target site, drug targeting systems also tend to be able to improve the efficacy of the drug (Moses *et al*, 2003; Torchilin, 2005; Duncan, 2006; Lammers *et al*, 2008). As a matter of fact, as depicted schematically in the lower two panels in Figure 1C, the implementation of a drug targeting system generally not only increases the concentration of the active agent at the target site, but it also improves its availability over time (i.e., its area under the curve, as a result of the enhanced permeability and retention (EPR) effect (Maeda *et al*, 2000)). When combining a clinically relevant regimen of (weekly) chemotherapy with a clinically relevant regimen of (daily) radiotherapy, it can therefore be expected that a drug delivered to the tumour by means of a drug targeting system interacts more effectively with radiotherapy than does a free, untargeted drug (Figure 1D). As will be outlined below, this was indeed found to be the case, and both for doxorubicin and for gemcitabine, it could be demonstrated that drug targeting systems increase the efficacy of radiochemotherapy without increasing its toxicity.

## Materials and methods

### Synthesis and characterisation of the copolymers

The tyrosinamide (TyrNH_2_)-, gadolinium (Gd)- and doxorubicin-containing *N*-(2-hydroxypropyl)methacrylamide (HPMA) copolymers were synthesised as described previously (Lammers *et al*, 2005; Kiessling *et al*, 2006). Details on the synthesis of these copolymers, as well as on the preparation of the two newly generated gemcitabine-containing copolymers are provided as Supplementary Information online. The weight- and number-average molecular weights (*M*_w_ and *M*_n_) and the polydispersity (*M*_w_/*M*_n_) of the copolymers were determined by size exclusion chromatography, and the amounts of doxorubicin and gemcitabine incorporated by means of spectrophotometry. The (weight-) average molecular weights of the two tyrosinamide-containing copolymers (i.e., poly(HPMA-co-MA-TyrNH_2_)) were 30.5 and 64.5 kDa, their polydispersities were 1.3 and 1.2, and the amounts of tyrosinamide, included to allow for radiolabeling, were 0.8 and 1.1 mol%. The average molecular weight of the gadolinium-labelled copolymer (i.e., poly(HPMA-co-MA-AH-Asp-[(Asp-(OH)_2_]_2_)-gadolinium) was 24.8 kDa, its polydispersity was 1.9 and the amount of gadolinium was 5.2 wt% (as determined by inductively coupled plasma mass spectrometry). The average molecular weights of poly(HPMA)-GFLG-Dox (i.e., PK1) and human immunoglobulin G-modified poly(HPMA)-GFLG-Dox (i.e., IgG-PK1) were 27.9 and 900 kDa, their polydispersities were 1.5 and 4.0, and relative amounts of doxorubicin were 6.5 and 4.8 wt%, respectively. The average molecular weights of A-Gem (i.e., poly(HPMA)-AH-Gem; uncleavable) and of B-Gem (i.e., poly(HPMA)-GFLG-Gem; cleavable) were 20.2 and 24.0 kDa, their polydispersities were 1.5 and 1.6, and their drug contents were 6.8 and 10.9 wt%, respectively.

### Drug release and *in vitro* efficacy of the Gem-containing copolymers

The release of Gem from A-Gem and from B-Gem was investigated at pH=7.4, at pH=6.0, and at pH=6.0 in the presence of the lysosomal cysteine protease cathepsin B. The temperature was set to resemble physiological conditions (i.e., 37°C). The concentrations of the two polymeric prodrugs were 2.3 × 10^−3^ mol l^−1^ Gem equivalent. The concentration of cathepsin B was 1.9 × 10^−7^ mol l^−1^, and its activity was standardised using the substrate *α*-*N*-benzoyl-DL-arginine *β*-naphthylamide. Drug release was quantified spectrophotometrically at 310 nm. The cytotoxicity of free and HPMA copolymer-bound gemcitabine was determined by seeding Dunning AT1 rat prostate carcinoma cells and A2780 human ovarian carcinoma cells into six-well plates, and by incubating them with increasing concentrations of the free drug, the two polymeric agents, and a drug-free control copolymer. Eight to ten days later, the cells were fixed and stained with crystal violet, and the number of surviving colonies was counted.

### Biodistributional analyses

The tumour and organ accumulation of the copolymers were evaluated by means of magnetic resonance imaging (MRI) and *γ*-scintigraphy. The former was performed using a clinical 1.5 T whole-body MRI system (Siemens Symphony) and a custom-made radio frequency (RF) coil (Kiessling *et al*, 2006). To optimise the signal-to-noise ratio, manual tuning and matching of the RF coil's circuitry was performed before each individual measurement. Additional details on the MRI-based biodistributional analyses are presented as Supplementary Information. For the scintigraphic analyses, two differently sized poly(HPMA-co-MA-TyrNH_2_) conjugates were radiolabeled with iodine-131 (by means of the Iodogen method). Labelling efficacies were always >95% (Lammers *et al*, 2006). Five hundred microliters of a saline solution containing 0.1 mM of the labelled copolymers (∼300 *μ*Ci) were subsequently injected i.v. into male Copenhagen rats. At several different time points p.i., blood samples were collected for kinetic analyses, and the biodistribution of the copolymers was visualised by means of a Searle-Siemens scintillation camera. At 24 and 168 h, animals were killed, and tumours and organs were harvested for quantification. The residual amounts of radioactivity were determined using a gamma counter, they were corrected for radioactive decay and they were expressed as percent of the injected dose per gram tissue.

### Therapeutic analyses

All experiments involving animals were approved by an external committee for animal welfare and were performed according to the guidelines for laboratory animals established by the German government. Experiments were performed on 6–12 month old male Copenhagen rats, using the syngeneic and radio- and chemoresistant Dunning AT1 prostate carcinoma model. Fresh pieces (∼10 mm^3^) of an AT1 donor tumour were transplanted subcutaneously into both hind limbs of the animals. Prior to treatment, tumours were grown for 6–8 days, until they reached an average diameter of 6 mm. Free doxorubicin was administered at its maximum tolerated dose (MTD), that is, at three doses of 2.5 mg kg^−1^ (days 1, 8 and 15). PK1 was applied at three (doxorubicin-equivalent) doses of 5 mg kg^−1^, and IgG-PK1 at both the 2.5 and 5 mg kg^−1^ regimen. Local external beam radiotherapy was delivered by means of the Siemens Gammatron S (cobalt-60 *γ*-irradiation; dose rate ∼0.5 Gy min^−1^) and it was applied at a regimen comparable to that routinely used in clinics: 2 Gy was given on every weekday for four consecutive weeks, i.e., for a total of 20 doses of 2 Gy (days 1–5, 8–12, 15–19, and 22–26). gemcitabine and the gemcitabine-containing copolymers were administered four times at a dose of 3 mg kg^−1^ (days 1, 8, 15, and 22). In this case, radiotherapy was delivered three times weekly for 4 weeks at a dose of 3 Gy, that is, for a total of 12 doses of 3 Gy (days −1, 1, 3, 6, 8, 10, 13, 15, 17, 20, 22, and 24). Tumour volumes were calculated using the formula *V*=(*a* × (*b* × *b*))/2, and they were expressed relative to the volume determined on the first day of therapy. The toxicity of the combination regimens was assessed by determining the body weight loss of the animals and by analysing the number of white blood cells, red blood cells, and platelets (Bayer Advia 120 hematology analyzer).

### Statistical analysis

Values are expressed as average±s.d. or as average±s.e.m. In the biodistributional analyses, the two-tailed *t*-test (for standard comparisons) or the paired *t*-test (for the left *vs* right comparisons) was used. In the therapeutic analyses, the Mann–Whitney *U*-test (i.e., Wilcoxon rank-sum) was used. Bonferroni–Holm *post hoc* analysis was used to correct for multiple comparisons. In all cases, *P*<0.05 was considered to represent statistical significance.

## Results

### Biodistributional analysis of HPMA copolymers

To assess the validity and the therapeutic potential of carrier-based radiochemotherapy, HPMA copolymers were selected as a model drug targeting system. Copolymers of *N*-(2-hydroxypropyl)methacrylamide (Supplementary Figure 1A) are prototypic and well-characterised polymeric drug carriers that have been broadly implemented in the delivery of anticancer therapeutics (Kopecek *et al*, 2000; Rihova and Kubackova, 2003a; Duncan, 2006; Lammers *et al*, 2008). Supplementary Figure 1B schematically depicts the different copolymers used in this study, functionalised, for example, with Gd and iodine-131 for imaging purposes, and with doxorubicin and gemcitabine for therapeutic purposes.

The biodistribution of the copolymers was evaluated by means of MRI, *γ*-scintigraphy and HPLC. The MR angiography scans in Figure 2A show that at 0.5 h post i.v. injection (p.i.), a 25 kDa gadolinium-labelled HPMA copolymer was localised predominantly to the vascular compartment. The color-coded maximum intensity projection (MIP; right panel in Figure 2A) confirms the long-circulating properties of the copolymer, showing that also in tumours, the targeting system still resided predominantly within the vasculature at this time point. Up to 10 h p.i., the tumour, kidney, and liver concentrations (i.e., whole organ levels) of the conjugate were then compared to those of Gd-DTPA-BMA (i.e., gadolinium-diethylenetriaminepentaacetic-acid-bis-methylamide; Omniscan®; 0.5 kDa), which is a prototypic low molecular weight MR contrast agent that does not bind to plasma proteins and that is rapidly eliminated from blood by means of renal filtration. As shown in Figures 2B and C, it was found that the implementation of the targeting system attenuated the renal clearance of the gadolinium label (reducing the initial peak in kidney accumulation from 60.1±7.5 to 28.5±5.0 *μ*M; *P*<0.05), and that it consequently – i.e., as a result of the EPR effect (22) – enhanced its accumulation in tumours over time. Significant localisation to liver was also noted (Figure 2C), but one needs to take into account that (I) the weight (and volume) of a rat liver tends to be ∼10 times higher than that of an average (10 × 10 mm) AT1 tumour, and that (II) at such ‘early’ time points, the signal quantified does not necessarily reflect contrast agent accumulation, as significant amounts of the copolymer are still present in circulation up to 24 h (see below), and as the liver is a highly vascularised organ.

To more extensively evaluate the tumour and organ accumulation of the polymeric drug delivery system, and to do so at later time points, we next radiolabeled two differently sized tyrosinamide-containing HPMA copolymers with iodine-131, and we monitored their biodistribution scintigraphically. In line with the MR angiography data Figure 2A, the images in Figure 3A on the one hand again demonstrate that the polymeric drug carriers circulate for prolonged periods of time, with especially for the 65 kDa copolymer, substantial amounts still present in blood at 0.5 and 24 h p.i. (as exemplified by the high levels localised to heart). Quantification of the concentrations of the two copolymers in systemic circulation confirmed this observation, with at 24 h p.i., for instance, 11.2±0.7 and 23.7±1.2% of the injected dose still present in blood for 31 and 65 kDa pHPMA, respectively (Figure 3B). The scintigrams in Figure 3A on the other hand also quite convincingly demonstrate that the polymeric drug delivery system presents with an acceptable biodistribution, with besides localisation to tumours, only indications for an accumulation in organs of the reticuloendothelial system (RES; i.e., liver, spleen, and lung), which is known to be involved in the clearance of long-circulating nanomedicines (Kopecek *et al*, 2000; Maeda *et al*, 2000; Torchilin, 2005; Duncan, 2006). In line with this, when quantifying the tumour and organ concentrations of the smaller copolymer at 24 and 168 h p.i., actually only in spleen, levels were always significantly higher than in tumours (Figure 3C). In lung, comparable levels were found, and in all other organs, the concentrations of the copolymer were significantly lower than in tumours. For 65 kDa pHPMA, an identical pattern was observed, the only difference being that the targeting efficacy appeared to be lower at 24 h p.i. and higher at 168 h p.i. (Figure 3C). This can be explained by taking the basic principles of EPR and the prolonged circulation time of the larger copolymer into account (Figure 3B), and is exemplified by the fact that levels in tumour and spleen substantially increased and levels in healthy tissues substantially decreased over time (Figure 3C). The tumour-to-organ ratios in Figure 3D confirm this observation, showing both higher overall values and larger increases over time for the 65 kDa copolymer, and they furthermore illustrate that HPMA copolymers localise to tumours relatively selectively, with throughout follow-up, always higher levels in tumours than in seven out of nine healthy tissues. Using HPLC, it was finally demonstrated that by means of their beneficial biodistributional properties, HPMA copolymers are able to improve the tumour-directed delivery of doxorubicin, increasing its target site accumulation at 24 h p.i. by more than a three-fold (22.1 *vs* 6.2 *μ*g doxorubicin per gram tumour; Supplementary Figure 2). Together, these findings show that HPMA copolymers are able to improve the temporal and spatial parameters of low molecular weight agents, and they thereby exemplify that HPMA copolymers are suitable targeting systems for assessing the validity and the therapeutic potential of carrier-based radiochemotherapy.

### Radiotherapy improves drug targeting

**Figure 1:**
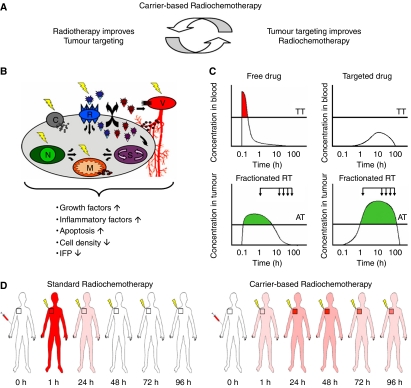
Rationale for carrier-based radiochemotherapy. (**A**) Carrier-based radiochemotherapy is based on the notion that drug targeting and radiotherapy interact synergistically, with on the one hand, radiotherapy improving the tumour accumulation of drug targeting systems, and with on the other hand, drug targeting systems improving the therapeutic index of radiochemotherapy. (**B**) Potential physiological mechanisms by which radiotherapy increases the tumor accumulation of drug targeting systems. See text for details. (**C**) Schematic representation of the blood and tumour concentrations of an intravenously (i.v.) applied free drug (upper and lower left panels), and of a drug delivered to the tumour by means of an i.v. applied drug targeting system (upper and lower right panels). The arrows indicate the administration of fractionated radiotherapy, which is routinely applied on every weekday for several consecutive weeks. TT: toxicity threshold, AT: activity threshold. See text for details. (**D**) Schematic representation of the *in vivo* interaction between radiotherapy and chemotherapy upon standard and upon carrier-based radiochemotherapy, exemplifying that in case of the latter, the temporal and spatial interaction between the two treatment modalities is improved.

To address the former aspect of carrier-based radiochemotherapy, that is, to evaluate whether radiotherapy is able to improve the tumour accumulation of drug targeting systems (Figure 1B), we next visualised and quantified the concentrations of the iodine- and gadolinium-labelled copolymers in tumours that were exposed to 20 Gy of radiotherapy 24 h before i.v. injection. The scintigrams in Figure 4A show that as hypothesised, ionising radiation indeed significantly improved the tumour accumulation of the carrier systems. Quantifications at 24 and 168 h p.i. confirmed this notion, showing that for the radiolabeled 31 kDa copolymer, increases of 24 and 57% were found (Figure 4B), and for the 65 kDa copolymer, increases of 46 and 48%, respectively (Figure 4C). Using the abovementioned 25 kDa gadolinium-containing copolymer and a 1.5 T clinical MR scanner, we subsequently also quantified the tumour accumulation of the targeting system at several earlier time points, that is, between 0.5 and 24 h after i.v. administration. In this case, upon quantifying the T1 signal enhancements in irradiated *vs* control tumours, increases ranging from 31 to 44% were observed (Figures 4C and D). Even though such increases may intuitively seem to be modest, in line with previous findings, using different doses, tumour models, and carrier systems (Li *et al*, 2000; Davies *et al*, 2004; Lammers *et al*, 2007), they do convincingly demonstrate that the tumour accumulation of drug targeting systems can be improved by combining them with radiotherapy. This in contrast, for instance, to the majority of targeting ligands that have been evaluated for this purpose over the years, but that generally only tend to improve the internalisation of the systems (Park *et al*, 2004; Kirpotin *et al*, 2006).

### Drug targeting improves doxorubicin-based radiochemotherapy

To address the latter (and clinically more relevant) aspect of carrier-based radiochemotherapy, that is, to investigate whether drug targeting systems are able to improve the interaction between radiotherapy and chemotherapy (Figure 1D), two different versions of HPMA copolymer-bound doxorubicin were synthesised, that is, poly(HPMA)-GFLG-Dox (PK1; 28 kDa) and human immunoglobulin G-modified poly(HPMA)-GFLG-Dox (IgG-PK1; 900 kDa). Both polymeric prodrugs have been tested in clinical trials, and both have been shown to be at least equally effective as free doxorubicin (Kopecek *et al*, 2000; Bilim, 2003; Rihova and Kubackova, 2003a; Rihova *et al*, 2003b; Duncan, 2006). As the MTD of PK1 is known to be 4–5 times higher than that of free doxorubicin (both in humans and in rodents (Yeung *et al*, 1991; Vasey *et al*, 1999)), in an attempt to simultaneously increase the efficacy and reduce the toxicity of the intervention, we chose to apply PK1 at three times 5 mg kg^−1^ (days 1, 8, and 15), that is, at twice the dose used for free doxorubicin, which has a cumulative MTD of 7.5 mg kg^−1^ in rats. IgG-PK1 was applied both at the 2.5 and at the 5 mg^−1^kg regimen. As shown in Figure 5A, in the aggressively growing and radio- and chemoresistant Dunning AT1 model, all three doxorubicin formulations were found to be significantly more effective than control, but the improvements were rather modest, and neither PK1 nor IgG-PK1 turned out to be better than the free drug. When combined with 20 daily doses of 2 Gy of local tumour radiotherapy, however, in line with our rationale (Figure 1), the two polymeric prodrugs did present with a significantly higher therapeutic index than did the free drug: PK1 applied at three 5 mg kg^−1^ doses and IgG-PK1 applied at three 2.5 mg kg^−1^ doses were both found to be substantially more effective than three 2.5 mg kg^−1^ doses of free doxorubicin (Figures 5B and C), and both were also significantly less toxic (Figure 5D). For the former two regimens, a dose-enhancement factor (DEF; i.e., a routinely used parameter for assessing the radiosensitising potential of chemotherapeutic agents) of 1.50 was found, as compared to a DEF of only 1.28 for the free drug (Supplementary Table 1). With a DEF of 1.82, IgG-PK1 applied at three 5 mg kg^−1^ doses was found to be by far the most effective regimen for improving the efficacy of fractionated radiotherapy (Figures 5B and C). It was, however, also the most toxic treatment (Figure 5D), which underlines the importance of determining an optimal dosing regimen for each individual agent. As it was found that both for PK1 (3 × 5 mg kg^−1^) and for IgG-PK1 (3 × 2.5 mg kg^−1^), the efficacy of the intervention was increased while its toxicity was reduced, these findings were considered to be an initial indication for the fact that drug targeting systems are able to improve the therapeutic index of radiochemotherapy.

### Preparation of gemcitabine-containing HPMA copolymers

To provide additional evidence for the validity of carrier-based radiochemotherapy, we subsequently synthesised pHPMA-Gemcitabine. Gemcitabine is a well-known radiosensitiser (Lawrence *et al*, 1997), and it is used in the first-line treatment of various advanced solid malignancies. Two different gemcitabine-containing copolymers were prepared, termed A-Gem and B-Gem (Figures 6A and B). In A-Gem, the drug is conjugated to the copolymer by means of the uncleavable aminohexanoic acid spacer. In B-Gem, gemcitabine is attached to the polymeric backbone by means of the -GFLG- spacer, which is also used in PK1 and which is known to be cleaved by the lysosomal cysteine protease cathepsin B. Figure 6C shows that A-Gem does not release the drug *in vitro*: independent of the conditions used, less than 1% of the agent was liberated upon 8 h of incubation. B-Gem, on the other hand, was quite effective in releasing gemcitabine: at pH=6, ∼10% was released after 8 h, at pH=7.4, ∼30% was released, and upon incubation with physiologically relevant concentrations of cathepsin B (at pH=6; resembling endo- and lysosomal conditions), the total amount of drug conjugated to the copolymer was released within less than 6 h (Figure 6D). To demonstrate that the extent of drug release correlates with the cytotoxicity of the conjugates, clonogenic survival assays were performed. As shown in Figures 6E and F, as expected, both in Dunning AT1 rat prostate carcinoma cells and in A2780 human ovarian carcinoma cells, B-Gem was significantly more effective than A-Gem. In addition, also in line with our expectations, the two carrier-based agents were found to be less effective *in vitro* than the free agent. For a drug-free control copolymer, no cytotoxicity was observed.

### Drug targeting improves gemcitabine-based radiochemotherapy

In the final set of experiments, we set out to investigate the *in vivo* potential of HPMA copolymer-bound gemcitabine. As shown in Figure 7A, it was again found that without radiotherapy, neither the free drug nor its polymeric prodrugs were able to induce substantial growth inhibition in the therapy-resistant Dunning AT1 model: B-Gem applied at four 3 mg kg^−1^ doses appeared to be the only regimen that was significantly more effective than control, but it was not more effective than free gemcitabine (Supplementary Table 2). In line with our rationale (Figure 1), however, upon again combining the agents with a clinically relevant regimen of fractionated radiotherapy (12 × 3 Gy), it could again be observed that the targeted formulation was significantly more effective than the free drug (Figures 7B and C): for B-Gem, a DEF of 2.79 was found, as compared to a DEF of ‘only’ 2.14 for free gemcitabine (Supplementary Table 2). Figure 7D and Supplementary Figure 3 finally show that the combination of B-Gem with fractionated radiotherapy was equally well tolerated as the combination of free gemcitabine with fractionated RT, with both agents inducing an identical degree of weight loss and of bone marrow suppression. In line with the results obtained for doxorubicin (Figure 5), these notions exemplify that drug targeting systems are able to increase the efficacy of radiochemotherapy without increasing its toxicity.

## Discussion

Over the years, a variety of different drug targeting systems have been developed, ranging in nature from simple polymers (Duncan, 2006) and liposomes (Torchilin, 2005), to stimuli-sensitive polymeric micelles (Rapoport *et al*, 2007), bacterially derived Minicells (MacDiarmid *et al*, 2007), and temporally targeted Nanocells (Sengupta *et al*, 2005). Thus far, however, not very many have managed to reach the final stages of clinical evaluation, and only about a handful have been approved by the responsible regulatory authorities. Consequently, hardly any information is available on the combination of tumour-targeted nanomedicines with other well-established treatment modalities. The potential of combining them with a clinically relevant regimen of fractionated radiotherapy, for instance, has not yet been properly evaluated, even though there is an obvious rationale for doing so (Figure 1).

**Figure 2:**
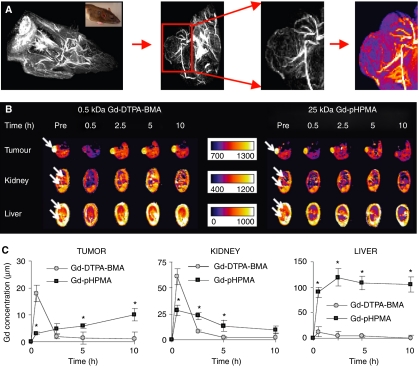
Magnetic resonance imaging (MRI)-based biodistributional analysis of pHPMA-gadolinium (Gd). (**A**) MR angiography scans of the chest and head region of a rat, of a tumour-bearing paw, and of an AT1 tumour, obtained at 0.5 h after the intravenous (i.v.) injection of 25 kDa pHPMA-Gd. A color-coded maximal intensity projection (MIP) of the polymer-visualised perfusion of the tumour is depicted in the right panel. (**B**) Dynamic, color-coded MRI T1 determination obtained for AT1 tumour, for kidney and for liver before contrast agent administration and at various time points after the i.v. injection of a low (0.5 kDa Gd-DTPA-BMA) and high (25 kDa Gd-pHPMA) molecular weight MR contrast agent. Note that contrast agent accumulation corresponds to a decrease in the T1 signal. (**C**) Quantification of the concentrations of gadolinium in AT1 tumour, kidney, and liver upon the i.v. injection of Gd-DTPA-BMA and pHPMA-Gd. Values represent average±s.d. (*n*=3). ^*^ Indicates *P*<0.05 (paired *t*-test).

**Figure 3:**
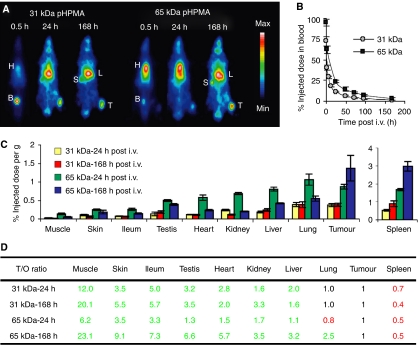
HPMA copolymers localise to tumours both effectively and selectively. (**A**) Scintigraphic analysis of the biodistribution of two differently sized iodine-131-labelled HPMA copolymers in Copenhagen rats bearing subcutaneously transplanted Dunning AT1 tumours, demonstrating prolonged circulation and effective tumour accumulation (H: heart (blood), B: bladder, S: spleen, L: liver, T: tumour). (**B**) Analysis of the blood concentrations of the two radiolabeled copolymers. Values represent average±s.d. (*n*=6). (**C**) Quantification of the tumour and organ concentrations of the two radiolabeled copolymers at 24 and 168 h post intravenous injection. Values represent average±s.d. (*n*=6). Except for lung and spleen, concentrations in tumours were always significantly higher than those in healthy organs (*P*<0.05; two-tailed *t*-test). (**D**) Quantification of the tumour-to-organ ratios of the copolymers analysed in (**C**), pointing out (in green) that they accumulate more selectively in tumours than in seven out of nine healthy tissues.

**Figure 4:**
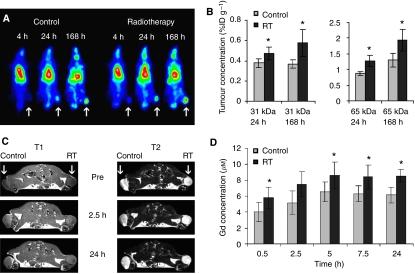
Radiotherapy (RT) improves drug targeting. (**A**) Scintigraphic analysis of the effect of 20 Gy of local tumour RT on the tumour accumulation of an iodine-131-labelled 31 kDa HPMA copolymer, demonstrating that RT beneficially affects tumour targeting. (**B**) Quantification of the effect of RT on the tumour concentrations of the 31 kDa (left panel) and 65 kDa (right panel) copolymer at 24 and 168 h post intravenous injection. Values represent average±s.d. (*n*=3–6). ^*^ Indicates *P*<0.05 (two-tailed *t*-test). (**C**) Magnetic resonance imaging analysis of the effect of 20 Gy of RT on the tumour accumulation of the 25 kDa gadolinium-labelled HPMA copolymer. The T1 images correspond to contrast agent accumulation, the T2 images were used for positioning and for morphological analysis. (**D**) Quantification of the effect of RT on the tumour accumulation of the 25 kDa gadolinium-labelled copolymer. Values represent average±s.d. (*n*=3). ^*^Indicates *P*<0.05 (paired *t*-test).

**Figure 5:**
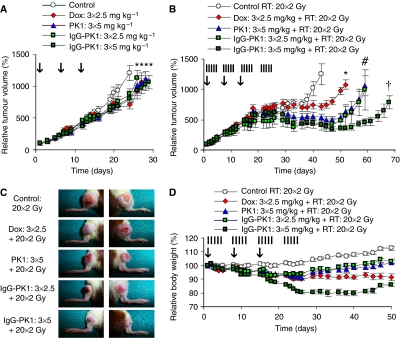
Drug targeting improves doxorubicin (Dox)-based radiochemotherapy. (**A**) Growth inhibition of Dunning AT1 tumours induced by three intravenous (i.v.) injections (days 1, 8, and 15; see vertical arrows) of saline, of free doxorubicin and HPMA copolymer-bound doxorubicin. PK1: pHPMA-GFLG-Dox (28 kDa). IgG-PK1: human IgG-modified pHPMA-GFLG-Dox (900 kDa). Values represent average ± s.e.m (*n*=6–12). ^*^Indicates *P*<0.05 *vs* control (Mann–Whitney *U*-test; Bonferroni–Holm *post hoc* analysis). (**B**) Tumour growth inhibition induced by three i.v. injections of the abovementioned chemotherapeutic agents in combination with a clinically relevant regimen of fractionated radiotherapy (20 × 2 Gy; see vertical lines). Values represent average±s.e.m. (*n*=8–10). ^*^ Indicates *P*<0.05 *vs* control, ^#^ indicates *P*<0.05 *vs* free Dox, and ^†^ indicates *P*<0.005 *vs* free Dox (Mann–Whitney *U*-test; Bonferroni–Holm *post hoc* analysis). (**C**) Representative images (day 50) of tumours treated with the indicated combination regimens. (**D**) Weight loss induced by doxorubicin-based combined modality therapy. Values represent average±s.e.m. (*n*=4–5).

**Figure 6:**
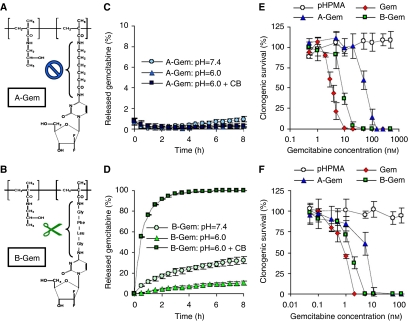
Characterisation of the gemcitabine (Gem)-containing HPMA copolymers. (**A**) and (**B**) Chemical structure of A-Gem (poly(HPMA)-AH-Gem) and B-Gem (poly(HPMA)-GFLG-Gem). (**C** and **D**) Release of gemcitabine from A-Gem and B-Gem at pH=7.4, at pH=6.0, and at pH=6.0 in the presence of the lysosomal cysteine protease cathepsin B (CB). Values are expressed relative to the total amount of drug conjugated to the copolymers, and they represent average±s.d. of three independent experiments. (**E** and **F**) Cytotoxicity of free gemcitabine, A-Gem, B-Gem and a drug-free control copolymer. Colony formation assays were performed using Dunning AT1 rat prostate carcinoma cells (**E**) and A2780 human ovarian carcinoma cells (**F**). Values represent average±s.d. (*n*=3).

**Figure 7:**
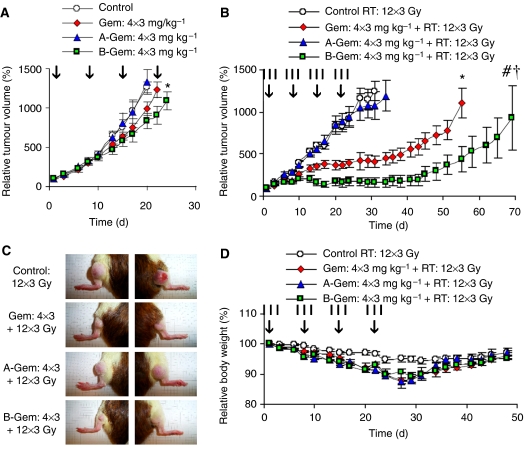
Drug targeting improves gemcitabine (Gem)-based radiochemotherapy. (**A**) Growth inhibition of Dunning AT1 tumours induced by four intravenous (i.v.) injections (days 1, 8, 15, and 22; see vertical arrows) of saline, free gemcitabine and HPMA copolymer-bound gemcitabine. A-Gem: pHPMA-AH-Gem (20 kDa). B-Gem: pHPMA-GFLG-Gem (24 kDa). ^*^ Indicates *P*<0.05 *vs* control (Mann–Whitney *U*-test; Bonferroni–Holm *post hoc* analysis). (**B**) Tumour growth inhibition induced by four i.v. injections of the abovementioned chemotherapeutic agents in combination with a clinically relevant regimen of fractionated radiotherapy (12 × 3 Gy; see vertical lines). Values represent average±s.e.m. (*n*=10–12). ^*^ Indicates *P*<0.005 *vs* control, # indicates *P*<0.0005 *vs* control, and ^†^ indicates *P*<0.05 *vs* free Gem (Mann–Whitney *U*-test; Bonferroni–Holm *post hoc* analysis). (**C**) Representative images (day 45) of tumours treated with the indicated combination regimens. (**D**) Weight loss induced by gemcitabine-based combined modality therapy. Values represent average±s.e.m. (*n*=5–6).

We have here used the prototypic polymeric drug carrier pHPMA to validate the potential of this targeted combination regimen. In line with the literature (Kopecek *et al*, 2000), HPMA copolymers were hereto first shown to be versatile and multifunctional drug carriers, that can be easily tracked *in vivo*, that circulate for prolonged periods of time, that localise to tumours both effectively and selectively, and that improve the tumour-targeted delivery of low molecular weight agents (Figures 2 and 3). Subsequently, they were then shown to interact synergistically with radiotherapy, with radiotherapy increasing the tumour accumulation of the copolymers (Figure 4), and with the copolymers increasing the therapeutic index of radiochemotherapy (Figures 5 and 7). Improvements were observed in a rapidly growing and therapy-resistant tumour model, and both for doxorubicin and for gemcitabine, together indicating that ‘carrier-based radiochemotherapy’ is indeed a promising approach for improving the efficacy of combined modality anticancer therapy.

This notion is in line with the results of a recently published phase I trial, in which 12 patients with localised oesophageal and gastric cancer were treated with the combination of poly(L-glutamic acid) (PGA)-bound paclitaxel (Xyotax; 6 doses; weekly) and fractionated radiotherapy (28 cycles; 1.8 Gy; daily), and in which four complete responses and an additional seven partial responses (with reductions in tumour size of more than 50%) were achieved (Dipetrillo *et al*, 2006). Prior to this trial, preclinical studies had already identified Xyotax as a highly potent radiosensitiser. When a single i.v. injection of Xyotax was combined with a single dose of radiotherapy, for instance, the dose required to produce 50% tumour cure (TCD_50_) could be reduced substantially, from 53.9 to 7.5 Gy (Milas *et al*, 2003). When radiotherapy was delivered as five daily fractions, the effect of Xyotax was even more pronounced reducing the TCD_50_ from 66.6 to 7.9 Gy. In a follow-up study in the same tumour model (i.e., C3Hf/KamLaw mice bearing ∼7 mm syngeneic OCa-1 ovarian adenocarcinomas), similar results were reported for Abraxane, that is, for albumin-based paclitaxel, which also beneficially combined both with single dose and with fractionated radiotherapy, and which did not increase normal tissue radiotoxicity (Wiedenmann *et al*, 2007).

In comparable analyses, Harrington *et al* (2000) have demonstrated that also liposomes hold significant potential for combination with radiotherapy. They combined both PEGylated liposomal doxorubicin and PEGylated liposomal cisplatin with both single dose (4.5 and 9 Gy) and fractionated (3 × 3 Gy) radiotherapy, and they showed that animals treated with carrier-based radiochemotherapy survived for significantly longer periods of time than did animals treated with standard radiochemotherapy. Davies *et al* (2004) recently confirmed and extended these findings, showing that PEGylated liposomal doxorubicin (Caelyx; Doxil) significantly improves the efficacy of both single-dose (8 Gy) and fractionated (3 × 3.6 Gy) radiotherapy, and that it does so, at least in part, by improving the penetration and the intratumoral distribution of the agent. In their studies on polymeric radiosensitisers, Li *et al* (2000) also observed that radiotherapy increases the tumour accumulation of passively targeted nanomedicines, attributing at least part of the supra-additively improved efficacy of PGA-paclitaxel (Xyotax) and radiotherapy to a radiotherapy-induced increase in tumour localisation. It is interesting to note in this regard that the overall improvement in the tumour concentration of PGA-paclitaxel was virtually identical to that observed here for HPMA copolymers, with as compared to sham-irradiated controls, increases ranging from 25 to 50%. Also in line with our findings, Li *et al* demonstrated that this radiotherapy-induced increase in tumour accumulation could already be observed almost immediately upon i.v. injection (i.e., at 1 h p.i. *vs* at 30 min p.i. here), and remains relatively constant over time. Together with Davies *et al*'s findings on the enhanced penetration and the improved intratumoral distribution of PEGylated liposomal doxorubicin in response to radiotherapy, these observations suggest that radiotherapy beneficially affects both arms of the EPR effect: on the one hand, by e.g. enhancing the expression of VEGF (Li *et al*, 2000; Park *et al*, 2001), it likely increases the permeability of the tumour blood vessels towards long-circulating nanomedicines, and on the other hand, by e.g. reducing the tumour cell density (Peschke *et al*, 1999) and the interstitial fluid pressure (Znati *et al*, 1996), it likely enhances their retention and their intratumoral distribution. Although clinically clearly less important than the improved therapeutic indices resulting from carrier-based radiochemotherapy, these improvements in EPR-mediated drug targeting to tumours provide an additional rationale for combining tumour-targeted nanomedicines with radiotherapy.

In summary, using prototypic polymeric drug carriers, two different imaging techniques and two different chemotherapeutic agents, we here demonstrate that drug targeting and radiotherapy interact synergistically, with radiotherapy increasing the tumour accumulation of drug targeting systems, and with drug targeting systems increasing the therapeutic index of radiochemotherapy. We extend previous efforts by attempting to generalise the concept of ‘carrier-based radiochemotherapy’, by implementing clinically relevant regimens of radio- and chemotherapy, and by directly comparing the radiosensitising potential of polymeric prodrugs to that of free chemotherapeutic agents. The results presented and the insights obtained strongly suggest that carrier-based radiochemotherapy holds significant potential for improving the treatment of advanced solid malignancies.
