# Supplementary material for: Image-guided and passively tumour-targeted polymeric nanomedicines for radiochemotherapy
Source: Br J Cancer. 2008 Sep 2;99(6):900–10. doi: 10.1038/sj.bjc.6604561 (PMC2538765; doi:10.1038/sj.bjc.6604561)
Supplement: Supplementary Information [file 6604561x1.doc]

**SUPPLEMENTARY INFORMATION**

**Supplementary Figure 1:** HPMA copolymers: Versatile and multifunctional drug carriers.

**(A)** Chemical structure of an HPMA (*N*-2-(hydroxypropyl)methacrylamide) monomer. **(B)** Schematic representation of a functionalized HPMA copolymer and an overview over the functional groups introduced. Gd: Gadolinium. TyrNH2-^131^I: Tyrosinamide-bound radioactive iodine. Dox: Doxorubicin. Gem: Gemcitabine. IgG: Human IgG (used as an unspecific targeting moiety; see Rihova and Kubeckova, 2003a). Note that different (combinations of) functional groups were used in different experiments.


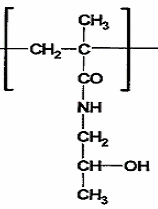

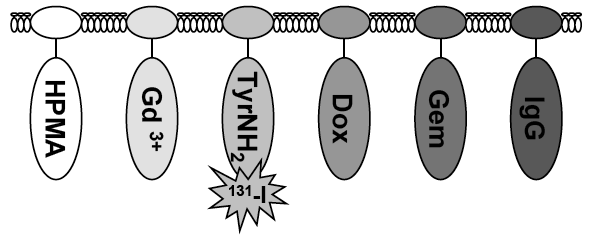


**A**

**B**

**Supplementary Figure 2**: Tumor accumulation of doxorubicin.

HPLC analysis of the μ doxorubicin concentrations in subcutaneous Dunning AT1 tumors 24 h after the i.v. injection of 5 mg/kg of free doxorubicin and 5 mg/kg doxorubicin-equivalent of PK1. Values represent average ± s.e.m. (n=4-6). * Indicates p<0.05 (two-tailed t-test).

*

**Supplementary Figure 3**: Hematological toxicity resulting from gemcitabine-based combined modality therapy.

The numbers of white blood cells (WBC), red blood cells (RBC) and platelets (PLT) were determined at several different time points after the start of treatment, and plotted relative to the number of cells at day 1. Values represent average ± s.d. (n=5-6). Note that the blood cell counts started to increase approximately one week after the last dose of chemotherapy (i.e. from day 29 onwards), and that there was hardly any difference in toxicity between free and HPMA copolymer-bound gemcitabine.

**PLT**

**RBC**

**WBC**

**Supplementary Table 1:** Summary of the details of the experiments assessing the efficacy of doxorubicin-based radiochemotherapy.

N represents the number of tumors per experimental group. The T10-time is the time needed by the tumors to reach ten times the volume determined at the first day of therapy. DEF is the dose enhancement factor, which is calculated by dividing the T10-time for the respective radiochemotherapy regimen by the T10-time for radiotherapy alone, and which is a routinely used parameter for assessing the radiosensitizing potential of chemotherapeutic agents. The p-values versus control and versus the free drug are determined by means of the Mann-Whitney U test.

* Indicates a significantly improved antitumor efficacy upon correcting for multiple comparisons using Bonferroni-Holm post-hoc analysis.

|  | **n** | **T10-time**  **(days)** | **DEF** | **P-value**  **(vs. Control)** | **P-value**  **(vs. Dox)** |
| --- | --- | --- | --- | --- | --- |
| **Control** | 12 | 20.7 | ― | ― | ― |
| **Dox: 3x2.5 mg/kg** | 8 | 26.0 | ― | 0.0016 * | ― |
| **PK1: 3x5 mg/kg** | 6 | 26.7 | ― | 0.0066 * | 0.75 |
| **IgG-PK1: 3x2.5 mg/kg** | 8 | 25.1 | ― | 0.0087 * | 0.28 |
| **IgG-PK1: 3x5 mg/kg** | 8 | 27.8 | ― | 0.00037 * | 0.76 |
| **Control: 20x2 Gy** | 10 | 39.5 | 1 | ― | ― |
| **Dox: 3x2.5 mg/kg + 20x2 Gy** | 10 | 50.5 | 1.28 | 0.0065 * | ― |
| **PK1: 3x5 mg/kg + 20x2 Gy** | 10 | 58.6 | 1.49 | 0.0010 * | 0.013 * |
| **IgG-PK1: 3x2.5 mg/kg + 20x2 Gy** | 8 | 59.1 | 1.50 | 0.0014 * | 0.021 * |
| **IgG-PK1: 3x5 mg/kg + 20x2 Gy** | 10 | 72.0 | 1.82 | 0.00016 * | 0.00050 * |

**Supplementary Table 2:** Summary of the details of the experiments assessing the efficacy of gemcitabine-based radiochemotherapy.

N represents the number of tumors per experimental group. The T10-time is the time needed by the tumors to reach ten times the volume determined at the first day of therapy. DEF is the dose enhancement factor, which is calculated by dividing the T10-time for the respective radiochemotherapy regimen by the T10-time for radiotherapy alone, and which is a routinely used parameter for assessing the radiosensitizing potential of chemotherapeutic agents. The p-values versus control and versus the free drug are determined by means of the Mann-Whitney U test.

* Indicates a significantly improved antitumor efficacy upon correcting for multiple comparisons using Bonferroni-Holm post-hoc analysis.

|  | **n** | **T10-time**  **(days)** | **DEF** | **P-value**  **(vs. Control)** | **P-value**  **(vs. Gem)** |
| --- | --- | --- | --- | --- | --- |
| **Control** | 10 | 17.4 | ― | ― | ― |
| **Gem: 4x3 mg/kg** | 10 | 20.0 | ― | 0.19 | ― |
| **A-Gem: 4x3 mg/kg** | 10 | 17.5 | ― | 0.53 | 0.075 |
| **B-Gem: 4x3 mg/kg** | 10 | 23.1 | ― | 0.011 * | 0.22 |
| **Control RT: 12x3 Gy** | 12 | 25.1 | 1 | ― | ― |
| **Gem: 4x3 mg/kg + 12x3 Gy** | 10 | 53.7 | 2.14 | 0.00017 * | ― |
| **A-Gem: 4x3 mg/kg + 12x3 Gy** | 10 | 25.6 | 1.02 | 0.72 | 0.0015 |
| **B-Gem: 4x3 mg/kg + 12x3 Gy** | 12 | 70.0 | 2.79 | 0.000032 * | 0.025 * |

**SuppLEMENTARY MATERIALS AND METHODS**

**Synthesis of the Monomers**

*N*-(2-hydroxypropyl)methacrylamide (HPMA) was synthesized by the reaction of methacryloyl chloride with 1-aminopropan-2-ol in dichloromethane in the presence of sodium carbonate. *N*-methacryloyl tyrosinamide (MA-TyrNH_2_) was prepared by the reaction of methacryloyl chloride with tyrosinamide in distilled water. 6-Methacrylaminohexanoic acid (MA-AH-OH) and *N*-methacryloyl glycyl-DL-phenylalanylleucylglycine (MA-GFLG-OH) were prepared by Schotten-Baumann acylation of the amino acids and oligopeptides with methacryloyl chloride in an aqueous alkaline solution. MA-GFLG-ONp was synthesized by the reaction of MA-GFLG-OH with 4-nitrophenol in the presence of *N*,*N'*-dicyclohexylcarbodiimide in tetrahydrofurane. 3-(6-Methacrylamidohexanoyl)-thiazolidine-2-thione (MA-AH-TT) and 3-(N-methacryloyl-GFLG)-thiazolidine-2-thione (MA-GFLG-TT) were prepared by the reaction of MA-AH-OH or MA-GFLG-OH with 4,5-dihydrothiazole-2-thiol in the presence of *N,N'*-dicyclohexylcarbodiimide and 4-(dimethylamino)pyridine.

**Synthesis of the Tyrosinamide- and Gadolinium-containing Copolymers**

Poly(HPMA-co-MA-TyrNH_2_) was prepared by solution radical copolymerization of the monomers HPMA and MA-TyrNH_2_ in methanol. The precursor for poly(HPMA-co-MA-AH-Asp-[(Asp-(OH)_2_]_2_) was prepared by alkaline hydrolysis of the copolymer poly(HPMA-co-MA-AH-Asp-[(Asp-(O-CH_3_)_2_]_2_), which was itself synthesized by solution radical copolymerization of the monomers HPMA and Ma-AH-Asp-[(Asp-(O-CH_3_)_2_]_2_, in methanol, using AIBN as an initiator. After hydrolysis, dialysis and lyophilization, the conjugate it was dissolved in distilled water, and gadolinium chloride was added. The pH of the solution was gradually increased to 6.0 (by addition of 0.1 M NaOH), and the excess of gadolinium was removed by dialysis, using a Visking dialysis tube with a cut-off size of 3.5 kD. Finally, the amount of gadolinium incorporated into the copolymer was determined by means of ICP-MS and the conjugate was lyophilized.

**Synthesis of the Doxorubicin-containing Copolymers**

The precursor for poly(HPMA)-GFLG-doxorubicin (PK1), i.e. poly(HPMA-co-MA-GFLG-ONp), was prepared by precipitation radical copolymerization of HPMA and MA-GFLG-ONp in acetone. The precipitated precursor was then filtered off, washed with acetone and diethyl ether, dried in vacuum, and conjugated to doxorubicin hydrochloride in DMSO, in the presence of triethylamine. The resulting polymer-drug conjugate was subsequently isolated by precipitation into a mixture of acetone-diethyl ether, filtered off, dried in vacuum, purified on a Sephadex LH-20 column in methanol (to remove free doxorubicin), and fractionated on a Sephadex LH-60 column (to obtain a narrow distribution of the molecular weight). For the preparation of IgG-poly(HPMA)-GFLG-doxorubicin (IgG-PK1), a comparable precursor, i.e. poly(HPMA-co-MA-GFLG-ONp), was used. This precursor, however, contained approximately twice as many ONp groups as the precursor used for synthesizing PK1, in order to allow for the binding of - besides doxorubicin - several IgG moieties. Upon the conjugation of doxorubicin, which was performed as described above, the precipitation-isolated intermediate with remaining ONp reactive groups was incubated with human IgG in saline-borate buffer (pH=8.0). Finally, the conjugate was purified by gel filtration on a Sephadex G-25 column and lyophilized.

**Synthesis of the Gemcitabine-containing Copolymers**

Polymer conjugates of gemcitabine have not been prepared thus far, and their synthesis will therefore be described in a bit more detail. Precursors containing thiazolidine-2-thione imide (TT) reactive groups (i.e. poly(HPMA)-co-MA-AH-TT for A-Gem, and poly(HPMA)-co-MA-GFLG-TT for B-Gem) were prepared by solution radical copolymerization of HPMA and MA-AH-TT, and of HPMA and MA-GFLG-TT, in DMSO, respectively. The comonomer concentrations in the polymerization mixture were 12.5 wt%, and the concentration of the initiator AIBN was 2.0 wt%. After polymerization, the copolymers were precipitated into an acetone-diethyl ether mixture (3:1), the solvents were filtered off and the precipitate was dried in vacuum.

The precursor used for preparing A-Gem, i.e. poly(HPMA)-co-MA-AH-TT (1 g; 5.52 x 10^-4^ mol of TT reactive groups), was dissolved in a glass ampoule containing 10 ml of dry pyridine. Gemcitabine hydrochloride (0.248 g; 8.27 x 10^-4^ mol) was added, the solution was mixed (i.e. bubbled with argon) and the ampoule was sealed. The ampoule was then incubated in a water bath at 50 ºC for 28 h. The copolymer with bound gemcitabine was isolated by precipitation into a mixture of acetone-diethyl ether (2:1), the solvents were filtered off and the precipitate was dried in vacuum. The remaining TT reactive groups were aminolyzed by the addition of 20 μl of 1-amino-propan-2-ol in DMSO. The resulting conjugate (i.e. A-Gem; poly(HPMA-AH-Gem)) was subsequently purified from unbound gemcitabine by gel filtration on a Sephadex LH-20 column in methanol and lyophilized.

In principle, B-Gem (i.e. poly(HPMA)-GFLG-Gem) was synthesized by an identical procedure: 1.0 g of poly(HPMA-co-MA-GFLG-TT) (4.60 x 10^-4^ mol of TT reactive groups) was dissolved in a glass ampoule containing 10 ml of dry pyridine and mixed with 0.207 g of gemcitabine hydrochloride (6.89 x 10^-4^ mol). The solution was then bubbled with argon, sealed and incubated at 50 ºC for 28 h. Subsequently, the precipitate was again isolated into a mixture of acetone-diethyl ether (2:1), it was dried in vacuum, the remaining TT reactive groups were aminolyzed using 1-amino-propan-2-ol, and the polymer-drug conjugate was purified from unbound gemcitabine by gel filtration on a Sephadex LH-20 column.

**MRI Analysis**

Magnetic resonance imaging was performed using a clinical 1.5 T whole-body MRI system (Siemens Symphony) and a custom-made radio frequency (RF) coil for RF excitation and signal reception. The RF coil was designed as a cylindrical volume resonator with an inner diameter of 83 mm and a useable length of 120 mm. To optimize the available signal-to-noise ratio, manual tuning and matching of the coil’s resonance circuitry was performed prior to each individual measurement.

For MR angiography, four rats were anaesthetized by inhalation using a mixture of isofluorane, N_2_O and O_2_. Their tail veins were catheterized using a 24 G indwelling cannula, which was connected to a syringe filled with ~1 ml of pHPMA-gadolinium (Gd concentration: 0.05 mmol per kg). Blood vessels in s.c. inoculated AT1 tumors were imaged at 30 min p.i. using a 3D gradient echo pulse sequence (3 FLASH; TR/TE = 28.0/8.6 ms, flip-angle = 70°, field of view = 12.8x30x50 mm^3^, matrix = 128x317x512, reconstructed voxel size = 100x98x98 μm^3^; 2 acquisitions). Vessels in the head and chest region were visualized using a different 3D sequence: TR/TE = 10.2/3.8 ms, flip angle = 70°, field of view = 30x55x110 mm^3^, matrix = 60x256x512, reconstructed voxel size = 500x215x215 μm^3^; 4 acquisitions.

For comparing the biodistribution of the 25 kD pHPMA-gadolinium conjugate to that of a clinically relevant low molecular weight MR contrast agent (gadolinium-DTPA-BMA; Omniscan®; 0.5 kD; Amersham Buchler), we first imaged the tumor localization morphology with a T_2w_-Turbo-spinecho sequence (T_2w_-TSE_i_; TR/TE/Turbo-factor = 4000ms/96ms/7). In addition, the pre- and post contrast (25 min p.i.) values of the longitudinal (T_1_) and the transversal (T_2_) relaxation time were measured in liver, kidney and tumor. At a slice thickness of 3 mm, the T_1_ times were determined with a Saturation-recovery turbo gradient echo pulse sequence (CPMG_i_; TR/TE/α/T_rec_ = 8.3ms/4.1ms/12°/53ms-9s), whereas the T_2_-measurements were performed with a 32-echo Spinecho sequence (TR/TE = 2000ms/23ms-360ms). In this case, the concentrations of the contrast agents were equivalent to 0.1 mmol gadolinium per kg. Dynamic contrast-enhanced MRI was performed using a 3D-FLASH sequence (TR/TE/α = 45ms/8.3ms/45°; spatial resolution: Δx = 0.4 x 0.4 x 0.4 mm³), and each 3D-FLASH measurement was followed by a T_1_-determination (as described above). The T_1_- and T_2_-values were calculated with an IDL-based software using a least-square fit algorithm. On the one hand, T_1_- and T_2_-values were determined pixel-wise, in order to create color-coded parameter maps, and on the other hand, for assessing the contrast agent accumulation in tumors, a T_1_-value was calculated for a region covering the whole tumor. For kidney and for liver, contrast agent accumulation was analyzed analogously. The change of the longitudinal relaxation rate, 1/T_1_, over time was converted into contrast agent concentration using the formula 1/T_1_ = 1/T_1,0_ + (r_1_ x C), and relaxivities (r_1_) of 21.3 mM^-1^s^-1^ for pHPMA-gadolinium and 4.1 mM^-1^s^-1^ for gadolinium-DTPA-BMA (24).

**HPLC Analysis**

Rats bearing 8-12 mm AT1 tumors were i.v. injected with 5 mg/kg of free doxorubicin or 77 mg of HPMA copolymer-bound doxorubicin (PK1; 28 kD; 6.5 wt-% doxorubicin). Exactly one day later, tumors were harvested, frozen in liquid nitrogen and homogenized in saline. The total amount of doxorubicin in tumors was determined upon hydrolysis of the homogenate with 2 M HCl at 50º C for 1 h (i.e. doxorubicinone; upon release from he copolymer). Free doxorubicinone was then extracted from the homogenate using chloroform, evaporated to dryness, dissolved in methanol, and subjected to HPLC analysis (Reverse Phase Column Chromolith® Performance RP-18e (Merck)). The amounts of doxorubicinone were analyzed using a fluorescence detector (RF10AXL; Shimadzu; excitation wavelength 484 nm; emission wavelength 560 nm), and they were quantified using a calibration curve.
